# Towards improving phenotype representation in OWL

**DOI:** 10.1186/2041-1480-3-S2-S5

**Published:** 2012-09-21

**Authors:** Frank Loebe, Frank Stumpf, Robert Hoehndorf, Heinrich Herre

**Affiliations:** 1 Department of Computer Science, University of Leipzig, 04103 Leipzig, Germany; 2 Department of Genetics, University of Cambridge, Cambridge CB2 3EH, UK; 3 Institute of Medical Informatics, Statistics and Epidemiology (IMISE), University of Leipzig, 04107 Leipzig, Germany

## Abstract

**Background:**

Phenotype ontologies are used in species-specific databases for the annotation of mutagenesis experiments and to characterize human diseases. The Entity-Quality (EQ) formalism is a means to describe complex phenotypes based on one or more affected entities and a quality. EQ-based definitions have been developed for many phenotype ontologies, including the Human and Mammalian Phenotype ontologies.

**Methods:**

We analyze formalizations of complex phenotype descriptions in the Web Ontology Language (OWL) that are based on the EQ model, identify several representational challenges and analyze potential solutions to address these challenges.

**Results:**

In particular, we suggest a novel, role-based approach to represent *relational qualities *such as *concentration of iron in spleen*, discuss its ontological foundation in the General Formal Ontology (GFO) and evaluate its representation in OWL and the benefits it can bring to the representation of phenotype annotations.

**Conclusion:**

Our analysis of OWL-based representations of phenotypes can contribute to improving consistency and expressiveness of formal phenotype descriptions.

## Introduction

In recent years, molecular biology has made significant progress in understanding the mechanisms underlying human disease. Several studies investigate disease mechanisms in animals that serve as models for humans [1]. In particular, the targeted modification of the genetic markup of these organisms provides a powerful means to investigate the molecular mechanisms associated with heritable diseases in humans [2]. Large-scale mutagenesis projects are now underway with the aim to characterize the outcomes of null-mutations for every gene in an organism. The observable characteristics of these modified organisms (their phenotypes) are represented in model organism databases and can be utilized to suggest candidate genes for diseases for which no molecular origin is currently known [3].

To standardize the terminology used in describing phenotypes, multiple species-specific phenotype ontologies were developed. For example, the Mammalian Phenotype Ontology (MP) [4,5] is used to characterize phenotypes in mice and other mammals, and the Worm Phenotype Ontology (WPO) [6] is used to characterize *C. elegans *phenotypes. The Human Phenotype Ontology (HPO) [7] describes phenotypes in humans and is applied for describing human diseases and individual patients.

To translate phenotypes across species and enable their comparison with human phenotypes and diseases, a syntax for phenotype decompositions has been developed [8-10]. In this syntax, phenotypes are represented by a combination of a quality and one or more entities. The entities represent the entities that are affected by a phenotype and are either physiological processes and functions (from the Gene Ontology [11]) or anatomical structures as represented by species-specific anatomy ontologies. The Phenotypic Attribute and Trait Ontology (PATO) [12] is an ontology of qualities which is used to describe *how *an entity is affected within a phenotype. Entity-Quality (EQ) based specifications of phenotypes have been developed for several species-specific phenotype ontologies [10], including the HPO [7], MP [4,5,13], WPO [6], and others, thereby integrating pre- and postcoordinated biomedical ontologies [10,14]. Recently, mechanisms became available to enable the automated translation of phenotypes across different species [3,10]. In these methods, ontologies are integrated through species-independent ontologies, and automated reasoning over the integrated ontologies enables the automated comparison of species-specific phenotype information across multiple species. This approach crucially relies on the formalization of phenotype information in ontologies and model organism databases. With the increasing application of ontologies for data analysis, improving the representation of phenotype ontologies has the potential to directly affect and advance scientific analyses and discoveries.

**Figure 1:**
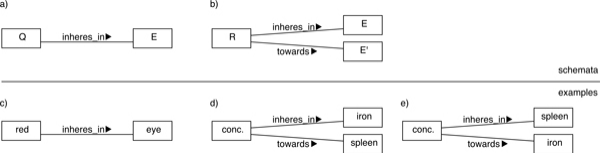
**EQ model**. In the schematic part, E stands for entity, Q for quality, and R for relational quality. Schema a) accounts for a simple (unary or non-relational) quality, while b) refers to relational qualities. The examples c) and d) correspond to those from the text. Example e) in parallel with d) is forestalling the problem of inter-modeler consistency from the Methods section. 'conc.' in d) and e) abbreviates *concentration of*.

The EQ model is an important and widely used means for formalizing phenotype information in ontologies [15]. In greater detail, its main idea is to combine an 'entity class' (the E in EQ) from an anatomy or process ontology with a 'quality class' (the Q) from PATO. For example, the class *eye *(MA:000261 in the Mouse adult gross anatomy ontology (MA) [16]) as the E and the color *red *(PATO:0000322) for Q can be combined to form the class *Red eye*. The typical formal interpretation of EQ statements is that the combination refers to a specialization of the quality class Q such that it inheres in instances of the entity class E [[10], p. 3], [17]. In the example, this yields the class *red that inheres in an eye *(cf. Figure 1, especially part c)).

Relational qualities involve at least one additional entity besides E. In the semantics of EQ, a second entity can be attached to a quality via the relation towards [[10], p. 3-5]. An example of this kind is the *concentration of iron in the spleen*, which can be formalized as a quality *concentration of *(PATO:0000033) inhering in *spleen *(MA:0000141) and connected via towards to *iron *(CHEBI:18248 in the ontology of Chemical Entities of Biological Interest [18]), in order to define *abnormal spleen iron level *(MP:0008739). Note that, despite continued use of this example, we will not go into detailed ontological analyses of the relationship between iron and spleen, e.g., as particulars/individuals. In particular, iron as an amount of matter/quantity or collection would deserve special treatment, cf. e.g. [19,20].

The term 'relational quality' as nowadays found in the bio-ontology community is typically used without further analysis, e.g. in [10], and via [17] can be traced back to [21] where it seems to be meant synonymously with the more widely used term 'relation'. Notably, in the context of formal ontology, by 'relational qualities' sometimes constituents of particular relation instances are referred to (in contrast to the overall relation instances themselves), termed 'relational roles' in section 'Enhancements for Relational Qualities' below.

While EQ descriptions characterize a phenotype, a related question pertains to the formalization of the *annotation *of organisms, genotypes and genes with EQ-based phenotype descriptions. In model organism databases such as the MGI database [13], genotypes like Add2*^tm1Llp ^*(MGI:2149065) are annotated with a class like *abnormal spleen iron level *(MP:0008739). The intended meaning of this annotation is that organisms of a particular mouse strain that exhibit the described genotype (a targeted mutation of the *Add2 *gene) within a specific environment will develop the *abnormal spleen iron level *phenotype. This complex relation can be simplified to improve performance of specific information retrieval tasks into a view in which the genotype is equivalent to the intersection of phenotypes, and individual mice are instances of their phenotypic annotations.

Only few efforts formally explore the compositional nature of phenotypes, i.e., how atomic phenotypes can be combined into more complex phenotypes such as in disease descriptions or in genotypes annotated with multiple phenotypes. In particular, the naive combination of phenotypes such as *red eye *with *short tail *is based on class intersections, and these lead to contradictory class definitions due to the disjointness of *color *(the super-class of *red*) and *size *(the super-class of *short*) [22]. More challenging are combinations of qualities which are hidden in the taxonomy of biomedical ontologies. For example, asserting that *red eye *is a sub-class of an *abnormal eye morphology *will imply that *red eye *is both a subclass of *morphology *and *color*. This will lead to another contradictory class definition due to the disjointness of *color *and *morphology *[23].

## Methods

### Identification of basic problems

We see three *basic problems *that need to be addressed regarding the representation of phenotypes and the interpretation of EQ descriptions in terms of the Web Ontology Language (OWL) [24], in order to utilize automated and semantically correct reasoning to its full extent.

I. ontological foundation of complex phenotypes

II. representation of phenotypes in formal languages

III. ontological foundation of phenotype annotations

The ontological foundation of complex phenotypes pertains to the problem of how combinations of concepts are correctly handled. It is argued that the current methods of combining concepts that are available in logical formalisms, including OWL, "have serious problems handling concept combinations in the way humans do." [[25], p. 19]. There the examples *tall squirrel, honey bee, stone lion*, and *white Zinfandel *are mentioned, for which no precise and correct representation within OWL is immediately available. Due to the connection with concept formation, the ontology of complex phenotypes exhibits an extensive and new research field that integrates methods and results of cognitive science and methods of formal ontology. Notably, in cognitive science itself research on conceptual combinations is still at a relatively early stage of development [26].

To address the first problem, we attempt to gain a clear understanding of the ontological nature of complex phenotypes and rely on an ontological framework for the explanation and foundation of complex phenotypes. This does not depend on the expressive power of OWL. Once we obtained an understanding of the ontological nature, this is the basis for investigating how to represent complex phenotypes in OWL, as a case of the second problem. The results of the first basic problem will influence the solutions to the second problem accordingly. Furthermore, limitations in expressiveness may cause the formal coverage of the ontological understanding to be partial, compared to the overall ontological picture.

The third step is to apply the theory to existing descriptions of complex phenotypes, such as those found in the phenotypic annotation of diseases and genotypes in model organism annotations.

### Focusing on issues of formal representation

As is clear from above, the first basic problem requires further attention, although it is already widely discussed, even in biomedicine and formal ontology, e.g. see [22,27,28]. In the present paper, our focus is on the second problem and its application to formalizing phenotype annotations. The latter, to a limited extent, also touches on the third basic problem. Regarding this focus, we identify five interrelated *particular issues *that affect our analyses.

1. ontological adequacy/coherence of ontological interpretation

2. invalid permutations/ambiguities

3. relational expressiveness

4. consistency of domain modeling

5. formal reflection of annotations

Referring to *ontological adequacy*, we intend to find OWL representations that are close to the ontological understanding of phenotypes as *qualities*, similar to established ontological theories of phenotypes [10,17]. While several approaches allow for representations of individual EQ statements in OWL, combining multiple EQ statements by means of their intersections may create incorrect [[22], sect. 4.2, p. 3117] and sometimes contradictory statements [23]. For instance, consider the following OWL concept:

(1) $$\left(\text{red}\;\text{that}\;\text{inheresIn}\;\text{some}\;\text{eye}\right)\;\text{and}\;\left(\text{short}\;\text{that}\;\text{inheresIn}\;\text{some}\;\text{tail}\right)$$

Concept (1) is necessarily empty, because no instance of red is equally an instance of short. Furthermore, this formalization faces the problem of *permutations *(issue two), arising from the commutativity and associativity of intersections in OWL. In particular, the parentheses in example (1) are merely auxiliary for reading. The concept is formally equivalent to (red that inheresIn some tail) and (short that inheresIn some eye). As a consequence, queries will deliver incorrect results if this mode of combining EQ statements is used.

The next two issues concern primarily phenotypes based on relational properties, like *iron concentration in the spleen. Relational expressiveness *is used for referring to limitations of the arity of relations that can be specified with an EQ description. The current model does not allow for relational qualities of an arity greater than two. This may lead to undesirable consequences, since several applications of biomedical knowledge representation require relations of higher arity [29,30]. This issue has been identified as a particularly important challenge for representing EQ-based phenotypes [17]. Closely connected to the number of arguments is the question of *inter-modeler consistency/harmonization*, cf. also [30]. This fourth issue refers to the question of how to link (a class representing) a relation to (classes of) its arguments such that it is as unambiguous as possible which argument connects to the relation in which way. In the current EQ model confusion can arise, e.g., on whether *iron concentration in the spleen *should be formalized as concentration that inheresIn some spleen and towards some iron or instead as concentration that inheresIn some iron and towards some spleen, see parts d) and e) of Figure 1. The different positions may correlate with the community/background of modelers, e.g. whether a biologist or a chemist makes the assertion. Corresponding decisions are not only relevant for formalization, but likewise influence querying. For the particular case of concentrations, [20] proposes inherence in those entities that are concentrated in another in the context of an ontological analysis, i.e., inherence in iron in the example. We comment on this in the discussion section below, with hindsight regarding our analysis.

The fifth and final issue is the orientation and clarification of how *annotations *are interpreted, for any account of phenotype representations. This immediately links back to the ontological reading of phenotype representations and the third basic problem above.

### Spectrum of solutions

In general, different approaches may be pursued in order to tackle the issues presented for the second basic problem. Like in [17], quality models that are fairly distinct from the EQ model may be (re-)considered. Another general change would be to concentrate on entities, i.e., primarily on the parts of an organism occurring in EQ descriptions, and to construct phenotype descriptions centering on them. E.g., the scheme *E *that hasQuality some *Q *follows this line of thought. Notably, the latter scheme is seen as equally eligible as phenotype description as the basic EQ scheme *Q *that inheresIn some *E *in [[17], sect. 2.3, p. 5]. Giving preference to the basic EQ scheme appears to have been an arbitrary choice. In terms of their relationship to annotated entities the two schemes differ evidently. Nevertheless, the entity-focused scheme shares analogous problems to those expounded for the basic EQ scheme, in particular the permutation problem.

In this paper we focus first on solutions that limit the number of changes to the established interpretation of EQ descriptions. The latter are meanwhile widely in use, cf. e.g. [15], as are phenotype ontologies with their basic presupposition of providing (sub)concepts of *quality*. Therefore, the migration to new proposals should be facilitated by an approach with less changes compared to more radical revisions.

## Results

### EQ interpretations with regard to annotations

First of all, what appears unavoidable is a more complex provision for annotations, at least if complex phenotypes formalized in OWL/description logics (DL) [31] shall be composable in terms of the usual intersection. Implicitly, this has already been observed in [22], to some extent also in connection with the EQ formalism. The following adheres to the understanding of annotations as outlined in the Introduction and is inspired by the notion of *phenes *in [22]. Nevertheless, the subsequent variant differs in order to minimize changes to PATO and phenotype ontologies.

In order to solve especially the permutation problem of combined EQ descriptions, formally it suffices to have an "encapsulating" relation available. For instance, while (1) suffers from unwanted permutations, this is avoided in (2), where the encapsulating relation is termed hasPheno.

(2) $$\begin{array}{c} \text{hasPheno}\;\text{some}\;\left(\text{red}\;\text{that}\;\text{inheresIn}\;\text{some}\;\text{eye}\right)\;\text{and}\\ \text{hasPheno}\;\text{some}\;\left(\text{short}\;\text{that}\;\text{inheresIn}\;\text{some}\;\text{tail}\right) \end{array}$$

**Figure 2:**
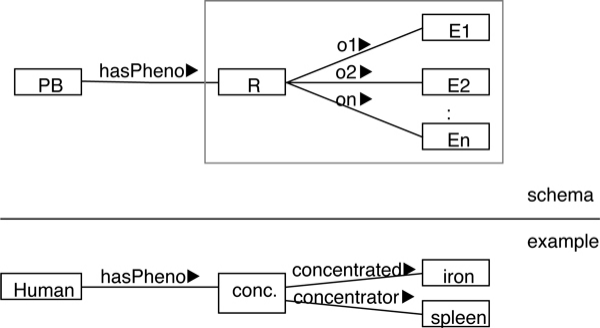
**Roles-as-properties: Ontological roles encoded as OWL properties**. PB stands for phenotype bearer in the schematic part.

**Figure 3:**
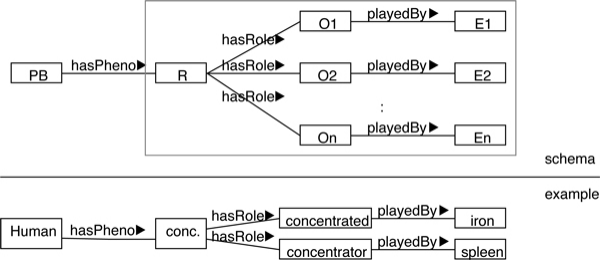
**Roles-as-classes: Ontological roles modeled as classes in OWL**.

Naturally, the question arises which ontological reading applies to hasPheno. We interpret (2) as a concept for classifying organisms (by two phenotype descriptions). The hasPheno relation belongs to an interpretive view/pattern that overlays common interconnections of entities, centering on the organism. In terms of the example, one may consider an organism *O *that has an eye *E *as its part, while there is a red *R *that inheres in *E*. Thus *O *is indirectly related with *R *in terms of common relations like inherence and part-of. In the phenotype view, this allows us to view *O*, as *phenotype bearer*, to exhibit *R as a pheno *of *O*. The latter connection is reflected by the hasPheno link between *O *and *R*. We require that each hasPheno link is "justified" by a chain of basic relations like *inheres-in*, *part-of, has-function, participates-in*, etc., that connects the entity in the pheno role with the one in the phenotype bearer role (PB in Figure 2, 3, 4 below). This approach leaves existing ontologies intact, resolves the first two particular issues identified, and accounts for the fifth, as well.

### Enhancements for relational qualities

#### Purely formal extension

On the remaining issues of relational expressiveness and consistency of domain modeling, we first observe that the current relational EQ model forms a special case of reifying (only binary) relations with *fixed *auxiliary relations, cf. the structural part of [32]. The main uncommon feature is the naming of those auxiliary relations as inheresIn and towards. One should admit, though, that inheresIn is meant to link to the ontological notion of inherence, whereas towards is introduced for rather technical reasons in [17] (circumventing an inherence relation of higher arity). It remains to be explored in greater detail whether towards can be adequately reinterpreted in terms of the notion of *external dependence*, see [[33], esp. sect. 6.2.7]. The more common approach to name those auxiliary relations would have been the use of names counting arguments, like argument_1_ and argument_2_. With the latter, an extension to *n*-ary relations is straightforward, which would solve the expressiveness issue. However, with fixed auxiliary relations there is no support for consistent domain modeling because the assignment of "values" to arguments is arbitrary. This may be the reason why all published variants of this pattern that we are aware of eventually suggest the *variable*, relation-specific naming of auxiliary relations [[29], sect. 5.1], [32,34].

Therefore, we do not see that changing the interpretation of relational EQ statements could be sidestepped, if inter-modeler consistent domain modeling is to be supported any further. Striving at the same time for ontological adequacy somewhat systematically, we adopt the model of relations and (relational) roles from the General Formal Ontology (GFO) [35,36], cf. also [37,38]. As a side remark, we indicate that there are more types of roles in GFO, but for brevity we use roles and relational roles as synonyms herein. Note further that from here on 'role' is reserved for the ontological interpretation, whereas the meaning as set of pairs/as binary relation in the context of description logics and OWL is referred to as 'OWL property' or 'DL role'.

#### Ontological alternatives using relations

In brief, relations in GFO are considered as categories of relators. *Relators *are ontological individuals akin to qualities, but with the power to mediate/connect entities. A relator consists of *role *individuals (via hasRole/roleOf) and each role individual, besides depending on the relator, depends on a *player *(via playedBy/plays). The term 'player' is relative to this approach; in general, arbitrary entities can play a role within a relation. At the categorial/class level, each relation *R *is associated with a set of role categories that forms the *role base *for this relation. Basically, that means for each relator of type *R *that its roles must instantiate one of the role categories in that set, cf. [[38], sect. 3.3.3].

The GFO model of relations and roles can be encoded into an OWL representation in two obvious ways, termed *roles-as-properties *(Figure 2) and *roles-as-classes *(Figure 3). Common to both cases is to represent phenotype descriptions involving a relation *R *and (kinds of) entities *E*_1_, . . . , *E_n _*as argument restrictions. Either, corresponding to Figure 2, roles are left implicit in the OWL properties *o*_1_, . . . , *o_n_*, or, regarding Figure 3, role categories are explicated as OWL classes *O*_1_, . . . , *O_n _*(in between *R *and the *E_i_*). Consider the example of *iron concentration in the spleen*, with the relation *concentration *and assuming that its two role categories are labeled *concentrated *and *concentrator*. The role of the *concentrated *is to be played by those entities that are concentrated in other entities, while the role of the *concentrator *is played by those (other) entities within which the first are concentrated. (Notably, in this technical reading *concentrator *must be understood merely like "container", which may be biologically misleading. In particular, anything in the *concentrator *role is *not *(necessarily) expected to cause or generate the concentration under consideration. A biological example of a concentrator in this non-intended sense could be a *kidney *that causes *urea *to be concentrated within the *bladder*. Despite this potential for confusion, *concentrator *is employed below in the "container"-sense in order to use a succinct role term that derives from the relation name.) The approach of roles-as-properties yields in OWL

(3) $$\begin{array}{c} \text{hasPheno}\;\text{some}\;\;\;\text{(}\;\text{concentration}\;\text{and}\\ \;\text{(concentrated}\;\text{some}\;\text{iron)}\;\text{and}\\ \;\text{(concentrator}\;\text{some}\;\text{spleen)}\;\;\;\text{),} \end{array}$$

whereas roles-as-classes leads to

(4) $$\begin{array}{l} \text{hasPheno}\;\text{some}\;\;\text{(}\;\;\text{concentration}\;\text{and}\\ \;\text{(hasRole}\;\text{some}\;\;\text{(concentrated}\;\text{that}\;\text{playedBy}\;\text{some}\;\text{iron)}\;\text{)}\;\;\text{and}\\ \;\text{(hasRole}\;\text{some}\;\;\text{(concentrator}\;\text{that}\;\text{playedBy}\;\text{some}\;\text{spleen)}\;\text{)}\;\;\;\text{)}\text{.} \end{array}$$

The first of these cases equals the above approach of using variable, relation-specific names for the auxiliary relations [[29], sect. 5.1], [32,34]. The second uses only two OWL properties hasRole and playedBy (and their inverses, possibly), but here this is unproblematic because the roles of the reified relation explicitly account for what is missing with fixed auxiliary relations without roles. Of course, both of these proposals will require a syntactic extension of the EQ model in order to capture the corresponding roles within EQ statements. Moreover, the roles-as-properties way may be simpler to reinterpret in other top-level ontological theories, because the roles presupposed by GFO are less explicit compared to roles-as-classes.

#### Ontological alternative using relations and qualities

The previous subsection suggests two ontologically inspired ways of formalizing relational qualities like *concentration of *(PATO:0000033, hereafter CO) in EQ statements that cure the immediate deficiencies previously described. We note that biological processes like concentrat*ing *anything in anything else are not taken into account here. Instead, both approaches are based on a purely relational reading of CO (and relational qualities, in general). CO is merely considered as a noun form of the phrase *is concentrated in *(CI). For example, '(a particular amount of) *iron I *is concentrated in a (particular) *spleen S*' is a "relational proposition", stating that *I *is concentrated in *S*. This proposition can be true or false, depending on whether the relation CI applies to *I *and *S *or not, but there is nothing to be measured, neither quantitatively nor qualitatively. (Pursuing this line of thought further in the example, one may wonder what remains as the actual difference between CI and relations such as 'is contained in' and 'is part of'.) In noun form, yet somewhat artificially, one may equivalently refer to 'there is concentration of *I *in *S*' for CI (note that *I *and *S *are particulars). However, we hold that CO comes in a second flavor, which is more amenable to specialization with notions like *increased concentration of *or to expressing specific values, e.g., 0.5_g_/*l*. In phrases like 'the concentration of *X *in *Y *is 0.5_g_/*l*', it appears more adequate to us to view CO as a proper quality which can be numerically quantified. Of course, immediately the question arises what that quality inheres in, which must be something that "includes" *X *and *Y*, not only one of the two. Here, computing the value of CO is instructive, which is based on values of qualities inhering solely in either *X *or *Y*, say, the weight of *X *and the volume of *Y*. The relationship between *X *and *Y *(of type CI, say) is characterized by the value within the CO phrase (in the second reading). Therefore, our current attempt of capturing relational qualities according to this analysis is to view them as inhering in particular relators, say a CI relator between *X *and *Y*. Admittedly, this is a deliberate, but no imperative choice among the possibilities within GFO. Other candidates for bearers of these qualities would be the overall relational fact, or one might consider the mereological sum of *X *and *Y*, in analogy to the inherence of relators in [[33], sect. 6.2.7]. If the latter option is to be followed, a more detailed analysis is required, though. Thinking of an amount of iron *I *concentrated in a spleen *S*, the question arises whether the mereological sum of *I *and *S *would differ from *S*. More generally, there may be interaction between the relation under consideration and forming a mereological sum of the relata. In any case, regarding implementation in OWL, we note that neither facts nor mereological sums are readily available on the basis of relators/relations and their arguments.

**Figure 4:**
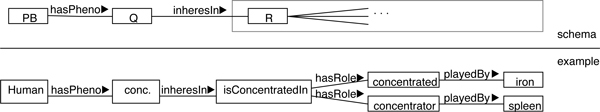
**Relator-based-quality: Relators characterized by qualities**.

Eventually we arrive at a third approach, depicted in Figure 4, where the relation is characterized by a quality. In the example, that means that CI is distinguished from CO, the latter being understood as a quality that inheres in CI relators/instances. Accordingly, we refer to this approach as *relator-based-quality*. Note that the intuitive term 'relational quality' experiences a formal-ontological reinterpretation from relations in the previous cases of roles-as-properties and roles-as-classes to qualities proper (which are not relations) in the relator-based-quality approach. Looking again at *iron concentration in the spleen*, assuming the roles-as-properties approach for modeling a relation isConcentratedIn (with roles like above) and a relational quality concentration yields in OWL

(5) $$\begin{array}{l} \text{hasPheno}\;\text{some}\;\;\text{(}\;\;\text{concentration}\;\;\text{that}\\ \;\text{(}\;\text{inheresIn}\;\;\text{some}\;\;\text{(}\;\text{isConcentratedIn}\;\text{and}\\ \;\text{(concentrated}\;\text{some}\;\text{iron)}\;\;\text{and}\\ \;\text{(concentrator}\;\text{some}\;\text{spleen)}\;\text{)}\;\text{)}\;\;\;\text{)}\text{.} \end{array}$$

This approach appears ontologically plausible to us currently, following the explanations above. Moreover, from the point of view of representation, it exhibits the beneficial property that CO is a "unary quality" like *color*, in the sense that it inheres in a single entity (a CI relator, which in turn accounts for the relational character of the quality). Any general account of representing measurements should thus be applicable to CO as it is to qualities like *color*. In these cases there is a single entity available - the quality - to which a measured value can be attached, where exploiting OWL datatype properties is one among several options that remain to be studied in future work. Furthermore, linking qualities to relators does not prescribe an overly specific relation model, but allows for adopting either of the approaches roles-as-properties and roles-as-classes in formalizing relations and roles, or even other theories (for which the quality bearer may require reinspection).

## Discussion

We limit the discussion mainly to aspects of the enhancements for relational qualities. Table 1 compactly summarizes the approaches that are considered herein. At the end, some remarks on measurements and comparative qualities are provided, and we briefly compare the relation hasPheno in this paper with has-phene from [22].

**Table 1:** Summary of the main features of the discussed approaches.

Feature		EQ	RP	RC	RQ
A	role information	no	yes	yes	yes
B	unlimited arity of relations	no (yes)	yes	yes	yes
C	variable arity of relations	no	yes	yes	yes
D	straight-forward database support	yes	partially	partially	partially
E	max. nr. of relevant vocabulary	2/0	0/*n *+ 1	2/*n *+ 1	*X *+ 1/*n *+ 2
F	add. characterization of relations	no	no	no	yes

Abbreviations of the approaches: EQ: entity-quality, RP: roles-as-properties, RC: roles-as-classes, RQ: relator-based-quality. The entry of '(yes)' in line B, column EQ, reflects the discussed extensibility of EQ. The numbers in line E count vocabulary elements (OWL properties or classes) introduced in each approach. The first number is the number of fixed elements (applicable to all relational qualities), in case of EQ this is two for inheresIn and towards. The second number is the maximal number of elements required per *n*-ary relational quality, e.g. *n *OWL properties encoding *n *roles and 1 OWL class encoding the relation itself for RP. The variable *X *in column RQ stands for the respective number of the RP or RC columns, depending on the relation model that is combined with RQ.

### Aspects of enhancements for relational qualities

In connection with the general annotation-oriented interpretation, all three approaches for an improved account of relational qualities are designed to satisfy the issues identified in the Methods section, possibly varying in their degree of ontological adequacy. Concerning major disadvantages, clearly, all cases lead to significantly greater complexity of the representation through a considerable extension of vocabulary elements (see Table 1 for details). Concerning the "style" of reification embodied in roles-as-properties and roles-as-classes, there are also further unintended *technical issues*, surveyed in [[30], sect. 2.2] (only with respect to roles-as-properties). At least in terms of reasoning, more precisely consistency checking and verifying entailments, those technical issues present no negative effects. Ibidem a number of potential *modeling shortcomings *are presented, in brief: (1) impeded manageability of the ontology, (2) purely technical nature of the additional vocabulary elements or at least an unclear ontological status, and (3) modeling diversity due to arbitrary splittings of reified relations, e.g. of reifying a 6-ary relation in terms of two ternary ones.

We disagree with all of these, yet to different degrees. Concerning (1), we agree that more vocabulary is involved which requires additional attention in *ontology maintenance*. But this can be countered by the mutual disjointness of relation, role, and non-relational classes and the use of distinct subsumption hierarchies/graphs for each category, within which relations, roles, and other classes can be organized manageably. Extra effort that remains is to determine role names for each relation when introducing the latter, which is a source of inter-modeler differences. One may adopt linguistic principles in some cases, supporting uniformity. For example, for binary relations that can be appropriately named by verbs, participles can be used as role names in many cases. Admittedly, that approach likely requires manual care and checking, e.g. remembering the remarks on misinterpreting the roles of *concentration of *(PATO:0000033) in the section Ontological alternatives using relations.

The *use of the ontology *may be less affected, if there are effective intermediate representations and user interfaces, cf. [[17], p. 1]. (2) is wrong in the light of the GFO approach to relations and roles, where these are ontological entities and thus not of purely technical nature. Admittedly, the roles-as-classes approach is closer to the ontological view of GFO, whereas roles-as-properties is a mainly technical simplification of the former. But this is not the technical nature criticized in [30]. Criticism (3) appears not applicable in our case, because the reification directly uses roles instead of arbitrary *k*-ary "parts" of an *n*-ary relation (where *k *<*n*).

Moreover, we see significant advantages in modeling and expressiveness that arise from the use of roles. For instance, relations are not only unconstrained in the number of arguments per relation, but one may even use anadic relations (i.e., with a variable number of arguments) and such with optional arguments. Similarly, symmetry properties of relations derive naturally from allowing for multiply instantiable role categories in the context of a role base. That means, a relation may be instantiated by relators that have several individual roles instantiating the same role category.

Notably, it is also symmetry of this kind that produces doubts on the treatment of concentration in [[20], sect. 3.2]. Hastings et al. present a fairly detailed analysis of substance mixtures (among other topics) which we can follow to a large extent. This analysis is aimed at formalizing the notion of concentration in description logics. In this connection and transferred to the original EQ model (cf. the Introduction and the Methods section), the consistency of domain modeling is achieved - for concentration only - by simply declaring that concentrations inhere in the entity, say iron, that is concentrated in another, say spleen. This likely means for EQ that the concentration is linked to that other entity by means of towards, and thus concentration that inheresIn some iron and towards some spleen is the preferred formalization, cf. the same example in the Methods section. At least, this is what we read from sect. 3.2 in [20]. It is not actually stated whether and how the concentration relates directly with a mixture, e.g. blood (in their example). One can also find more informal statements which suggest different interpretations, e.g. in sect. 2.2 of [20]. In any case, however, in their analysis this choice of assigning inherence and towards is not explained. Considering other relational properties than concentration, an analogous decision would have to be made for each relational property (and established among modelers), which appears less attractive than finding more general rules. Closing the circle to symmetric relations, for these it is not possible to distinguish one of the arguments (at least, not based on their roles only). For instance, for a phenotype like *increased distance of the eyes*, it appears completely implausible to select one eye in which a distance inheresIn, whereas it is towards the other eye. Especially the relator-based-quality approach, despite its own unresolved choices (see the respective part of the Results section), avoids such arbitrary fixing.

A practical factor of all three approaches that might be of potential importance is a slightly increased complexity, which prevents a straight-forward integration of corresponding annotations into the relational schemas of annotation databases. Relational database tables are set up with a fixed number of columns. While for the established EQ approach one may simply add one column for each of 'quality', 'entity-1', and 'entity-2' (and possibly for 'qualifier'), a role-based approach requires a more exible solution that allows for a variable number of entities as arguments of relations, for example. Thus we propose *not *to encode roles in the database schema, but rather as instance data (i.e., role-based information is captured row-wise rather than column-wise). Table 2 shows one corresponding proposal by way of example (jointly with Tables 3 and 4), yet more work in this direction remains to be done. To some extent, there seems to be an in-principle incompatibility of various aims, including the provision for *n*-ary relations vs. a simple database implementation.

**Table 2:** Sample phenotype table for annotation databases.

Phenotype Table					
***r_id***	***p_id***	***quality***	***role***	***entity***	***qualifier***

0	0	red PATO:0000322		eye MA:000261	
1	1	concentration of PATO:0000033	concentrator *N/A*	spleen MA:0000141	abnormal PATO:0000460
2	1	concentration of PATO:0000033	concentrated *N/A*	iron CHEBI:18248	abnormal PATO:0000460
3	2	increased concentration PATO:0001162	concentrator *N/A*	spleen MA:0000141	
4	2	increased concentration PATO:0001162	concentrated *N/A*	iron CHEBI:18248	

Tables 2-4 form an integrated set of database tables, as an example of one implementation option for annotation databases. The present table stores pure phenotype descriptions. It supports non-relational and relational qualities, in the latter case of arbitrary arity and role type. For this purpose, role types do not correspond to columns (attributes) of the table, but they are stored in rows. Rows of a single phenotype description have the same phenotype identifier assigned to them. The sample entries repeat examples of the main text (with natural language terms for readability), but are arbitrary otherwise.

Abbreviations: *r_id: *row identifier, *p_id*: phenotype identifier, *N/A*: not available. Empty positions are to be read as null values.

**Table 3:** Sample entity table for annotation databases.

Entity Table		
*e_id*	. . .	. . .

0	. . .	. . .
1	. . .	. . .
2	. . .	. . .
3	. . .	. . .

The database table for the description of entities to be annotated with phenotypic information can adhere to an arbitrary schema in the context of Tables 2-4. Only entity identifiers must be presumed for Table 4. This entity table is merely shown for completeness.

Abbreviation: *e_id*: entity identifier.

**Table 4:** Sample annotation table for annotation databases.

Annotation Table (for phenotypic annotations)			
***e_id***	***p_id***	***measured_value***	***measuring_unit***

0	0		
0	1	5	mg/kg
1	2		
2	2		

Assuming phenotypes and entities according to Tables 2 and 3, the actual annotations of entities with phenotypes are kept separately in this table, since this is a many-to-many-relationship. The two additional columns shall indicate a simple option to capture quantitative data. Notably, the qualifier column of Table 2 is placed therein for coherence with current EQ descriptions. That column may alternatively be part of the present table.

Abbreviations: *e_id*: entity identifier, *p_id*: phenotype identifier. Empty positions are to be read as null values.

Another interesting observation arises from considering the proposed database tables in Tables 2, 3, 4 in connection with the OWL representation schemes in Figure 2, 3, 4. Annotations in a corresponding database would be compatible with all approaches discussed above, because these rely on the same core of conceptual primitives. Database contents can thus be translated into OWL according to any of the three schemes. Consequently, the support of annotation databases provides no criterion to distinguish among the three approaches.

The remaining question may be: which approach should one favor most - roles-as-properties, roles-as-classes, or relator-based quality? Yet an answer crucially depends on the purpose and possibly the runtime behavior of those OWL representations. Further analysis will be necessary in both regards. Ignoring these aspects and adopting a merely descriptive point of view, we note that relator-based-quality (with roles-as-classes for capturing relations) relies on the most detailed analysis thus far and suggests a uniform manner of interfacing with representations of measurements for non-relational and relational qualities.

### Contextual aspects: measurements, qualifiers, and phenes

Having just mentioned the representation of measurements, this connects in a further way to the more general topic of relations (and phenotypes). The question arises whether the notion of an ontological relation (based on relators, as included in GFO) covers all relevant interpretations of the term 'relation' that are needed to construct and analyze all kinds of complex phenotypes. There is a reading of 'relation' expressed by comparative conditions like *X is redder than Y*, *X is faster than Y*, and *X is heavier than Y*. The specification of these relations assumes an ordering or scale on the instances of the properties *red*, *velocity*, and *weight*. Such a scale is in many cases the result of measuring, where a measure connects properties of the real world with numbers. The latter are included in the mathematical realm, and the orderings of numbers are usually considered as set-theoretic relations, i.e., as sets of ordered tuples. Based on the measuring process, these relations are transferred to relations between properties (e.g., instances of *red*, *velocity*, and *weight*). Accordingly, we expect that set-theoretic relations are likewise necessary to get a full picture of understanding the term 'relation' in the context of phenotype analysis and representation (and yet in other connections). We emphasize that GFO is an appropriate means for this analysis, since it includes both, relator-based relations and set-theoretic relations.

Next, let us briefly return to the hypothetical annotation database in Tables 2, 3, 4, where the phenotype table comprises a column named 'qualifier' (Table 2). This stays closely in line with the use of a *qualifier *(also called modifier) in EQ statements, indicated in [[10], p. 3 and Tables 2 and 4]. Actually, however, we regard a more comprehensive treatment of qualifiers as future work. It remains debatable, for instance, why abnormality (*abnormal*, PATO:0000460) is captured by means of the qualifier construct, whereas prefixes such as *increased *and *decreased *(e.g. in *increased concentration*, PATO:0001162) are parts of names of qualities. The treatment of 'qualifier' in Table 2 is therefore preliminary and the overall approach of encoding qualifiers may experience revision. Notably, implicitly comparative qualities such as *increased concentration *are usable with all approaches above in exact analogy to the their use with the EQ model. A final note concerns the hasPheno relation introduced in the Results section above. It is inspired by, but deviates from the notion of *phenes *and the has-phene relation in [22]. Therein, phenes may be understood as quality-like entities that reflect/abstract complex aspects that an organism is involved in. For instance, a structural phene accounting for *presence of a spleen *can be given the following class definition in OWL: present-spleen equivalentTo: phene-of some (has-part some spleen). This phenotype description can then be applied to an entity (class) in terms of the *has-phene *relation, e.g. human that has-phene some present-spleen. As a result of the logical constraints of the approach to phenes in [22], an instance *I *of the latter class must have a spleen as part, and *additionally *there must be a phene *P *(an attributive entity, different from the spleen) such that *I *has-phene *P*. The same applies in the case of qualitative phenes, e.g. cf. being-red equivalentTo: phene-of some (has-quality some red). Again, if a particular entity has a quality of type *red*, it also has a phene of type *being red*. Here lies the difference to the hasPheno approach above, which does yield new relational links between instances in addition to more basic relations, like *inherence, part-of*, etc. But it does not lead to additional instances, quality-like or otherwise, cf. the passage on EQ interpretations of the Results section. This suffices for our purposes, such that avoiding additional entities may be judged to be preferable. On the other hand, further comparison of the strengths and weaknesses of both views is a future task.

## Conclusions

In this paper we report on the (work-in-progress) state of our analyses and improvement proposals concerning the Entity-Quality (EQ) model. A simple general modification in the understanding of qualities in PATO is argued to be necessary. Moreover, three variants of formalizations of extended support for relations/relational qualities in the Web Ontology Language (OWL) are presented.

Much work remains to be done or completed. The approaches detailed herein rely on theoretical analyses thus far. For further assessment, an experimental evaluation should be conducted, e.g. exploring the efficiency of reasoning over ontologies which rely on one or another approach. Despite our (preliminary) decision to minimize changes to the EQ interpretation to the greatest possible extent, we still see many interesting open theoretical issues in the EQ model, respective ontologies, and phenotype understanding and representation in general. For instance, we are convinced that not all concepts of PATO should be regarded ontologically properly as qualities. The connections between hasPheno above and has-phene in [22] are only initially elaborated yet and deserve continued treatment, as well. Accordingly, further alternatives, which possibly involve larger reinterpretation of existing resources, should be studied and compared. On that basis EQ syntax extensions and possibly changes to phenotype ontologies can be devised.

## List of abbreviations

CHEBI: Chemical Entities of Biological Interest; CI: is concentrated in (relation name); CO: concentration of (relation/quality name); DL: description logic; EQ: Entity-Quality; GFO: General Formal Ontology; HPO: Human Phenotype Ontology; MA: Mouse Adult Gross Anatomy Ontology; MGI: Mouse Genome Informatics (database); MP: Mammalian Phenotype Ontology; OWL: Web Ontology Language; PATO: Phenotypic Attribute and Trait Ontology; WPO: Worm Phenotype Ontology.

## Competing interests

The authors declare that they have no competing interests.

## Authors' contributions

RH identified problems of the EQ model and initiated the work. FS and FL devised the modeling alternatives for relational qualities and determined their properties, with further support from RH. HH supervised the project. All authors wrote the paper, discussed and revised it. All authors read and approved the final manuscript.
